# Hybrid Strategy of Two-Level Cervical Artificial Disc and Intervertebral Cage: Biomechanical Effects on Tissues and Implants

**DOI:** 10.1097/MD.0000000000002048

**Published:** 2015-10-30

**Authors:** Tzu-Tsao Chung, Dueng-Yuan Hueng, Shang-Chih Lin

**Affiliations:** From the Graduate Institute of Applied Science and Technology (T-TC); Graduate Institute of Biomedical Engineering, National Taiwan University of Science and Technology (S-CL); and Department of Neurological Surgery, Tri-Service General Hospital, National Defense Medical Center, Taipei, Taiwan (T-TC, D-YH).

## Abstract

This numerical study aimed to evaluate tissue and implant responses to the hybrid surgery (HS) of cervical artificial disc replacement (C-ADR) and anterior cervical discectomy and fusion (ACDF).

Four hybrid strategies of two-level C-ADR and ACDF were compared in terms of adjacent segment degeneration (ASD) and implant failure.

The rotary C-ADR and semirigid ACDF have been extensively used in the multilevel treatment of cervical instability and degeneration, but the constrained mobility at the ACDF segments can induce postoperative ASD problems. Hybrid surgery of C-ADR and ACDF has been an alternative to provide the optimal tradeoff between surgical cost and ASD problems. The biomechanical effects of hybrid strategies warrant thorough investigation for the two-level instrumentation.

Based on computed tomography imaging, a nonlinear C2–C7 model was developed and validated by cadaveric and numerical data. Four strategies of inserting the C-ADR and ACDF into the C4–C6 segments were systematically arranged as PP (2 peek cages), AA (2 artificial discs), PA, and AP. The biomechanical behavior of these 4 strategies was evaluated in terms of motion and stresses of discs, facet forces, stresses of C-ADR and ACDF, and C-ADR motion.

The constrained mobility of the ACDF segment worsened the kinematic and mechanical demands of the adjacent segments and artificial discs. The C-ADR articulation provided higher mobility than the replaced disc of the intact construct, making it an effective buffer to accommodate the compensated mobility and load from the ACDF segment. Consequently, the ASD progression of the AA construct was most restricted, followed by the PA, AP, and PP construct.

The PA strategy is a tradeoff to preserve mobility and reduce cost. The C-ADR of the PA construct preserves the mobility of the C5/C6 segment and shares the transferred motion and loads of the fused C4/C5 segment. The PA construct shows optimal biomechanical results for minimizing ASD and implant failure, whereas the AP strategy is only recommended when cranial degeneration is the major concern.

## INTRODUCTION

After multilevel anterior cervical discectomy and fusion (ACDF), the accelerated degeneration of adjacent segments has been extensively reported as a fusion-related problem (ie, adjacent segment degeneration [ASD]).^1–3^ In the literature, the ASD problem has been numerical and experimentally demonstrated as the compensated result of fusion to adjacent segments.^4,5^ Consequently, the artificial disc has been developed as an alternative to provide the mobility of the instrumented segments and decrease the compensated mobility to the adjacent segments.^6,7^ The hybrid use of ACDF and cervical artificial disc replacement (C-ADR) has also been adopted to treat the multilevel instability and degeneration of the cervico-thoracic column.^8–11^ Theoretically, there are 4 strategies of two-level instrumentation to place the peek cage (P) and artificial disc (A): PP, AA, PA, and AP. The 2 continuous segments of the PP and AA strategies are instrumented with 2 peek cages and 2 artificial discs, respectively. For the PA strategy, a peek cage is placed at the cranial segment, followed by an artificial disc. The reverse placement is used for the AP strategy.

From the biomechanical viewpoint, the kinematic characteristics of the semirigid ACDF and rotary C-ADR represent the different rationales of implant design, namely fusion and dynamization, respectively. The hybrid use of the semirigid and dynamic implants potentially affects the construct behavior of multilevel instrumentation.^12–15^ Numerous follow-up studies have shown definite differences in the subsequent ASD progression between ACDF, C-ADR, and their hybrid use.^2,3,5,10,13^ The results consistently reveal that the C-ADR segment provides a mobility-buffering ability to the adjacent fused segment, thereby slowing the ASD progression from the fused to the adjacent segments.

However, the use of 2 C-ADRs is costly for most patients. In contrast, the PA and AP strategies can serve as tradeoff treatment between ASD progression and surgical cost. The current authors hypothesize that the decision-making factors between the PA and AA involve initial conditions of the instrumented and adjacent segments. To date, no study has systematically clarified how the initial conditions affect the surgical results of the 4 strategies. This was the objective of the current study.

Using the finite-element method, this study investigated the biomechanical differences between the PP, AA, PA, and AP placements for the hybrid surgery. Based on computed tomography (CT) imaging, a cervico-thoracic model with physiologic loads and degenerative segments was developed and validated in terms of cadaveric and numerical studies. The tissue responses and implant behaviors of intact and PP constructs were chosen as the comparison baselines to evaluate the biomechanical properties of other strategies. The findings were correlated with the clinical results found in the literature to gain insights into the cervical HS of C-ADR and ACDF.

## MATERIALS AND METHODS

### Development of a Cervical Model

**FIGURE 1:**
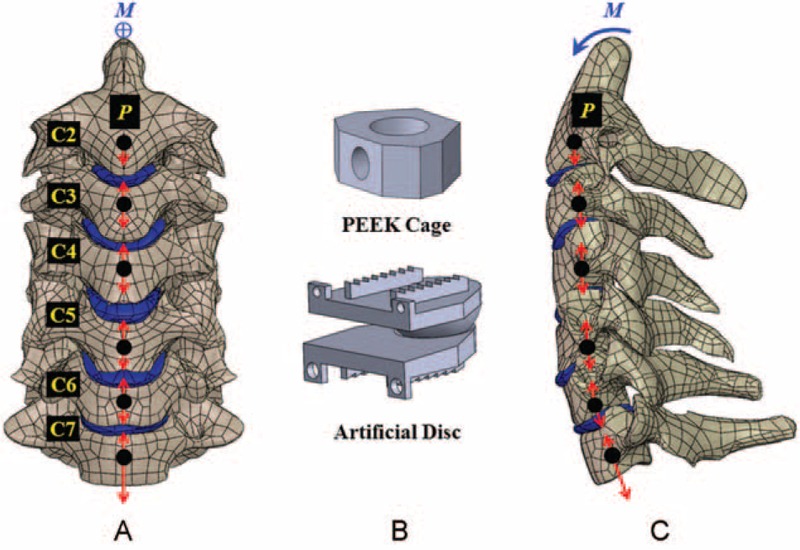
A nonlinear cervical model from C2–C7 segments is subject to ligament interconnection, follower loads (P), and cranial moment (M). A, Front view. B, Two static and dynamic implants. C, Lateral view.

The osseo-ligamentous C2–C7 model consisted of vertebral bones (anterior bodies, posterior elements, and endplates), intervertebral discs (annulus fibrosus and nucleus pulposus), and the surrounding ligaments (Fig. 1). The bony tissues of a cervical column were three-dimensionally reconstructed from the CT images of a healthy 55-year-old male subject without any disease. An anterior body was composed of a cortical shell and a cancellous core. The paired facet joints of the posterior elements were cautiously prepared to ensure interfacial contact during excessive motion. The curved gaps of the healthy facet joint were consistently 0.5 mm in the unloaded neutral position.^16^ The endplate was modeled as a plate of uniform 1-mm thickness and sandwiched between anterior body and intervertebral disc. Except for the cancellous core, the constitutive laws of all bony tissues were assumed to be linearly elastic and isotropic (Table 1).

**TABLE 1:**
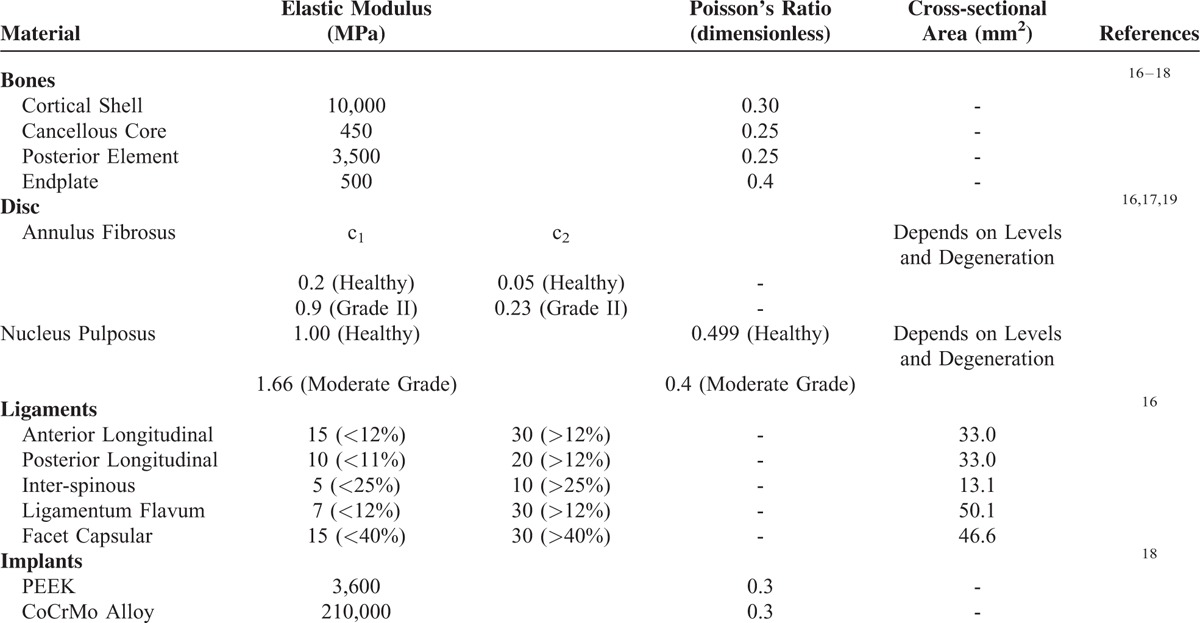
Material and Characteristics of Cervical Tissues and Implants

Based on the indefinite images and anatomical landmarks, the outlines of the fibrous tissues were manually developed by the software SolidWorks, Ed. 2013 (SolidWorks Corporation, Concord, MA). The annulus fibrosus and nucleus pulposus were simulated as hyperelastic composite and a cavity filled with noncompressive fluids, respectively.^17^ There were 7 ligaments included and modeled as tension-only springs to join their attachment points on adjacent vertebrae (Table 1). The mechanical properties of all tissues were cited from literature data and their shapes were assumed to be symmetrical with respect to the sagittal plane.^16–18^

**FIGURE 2:**
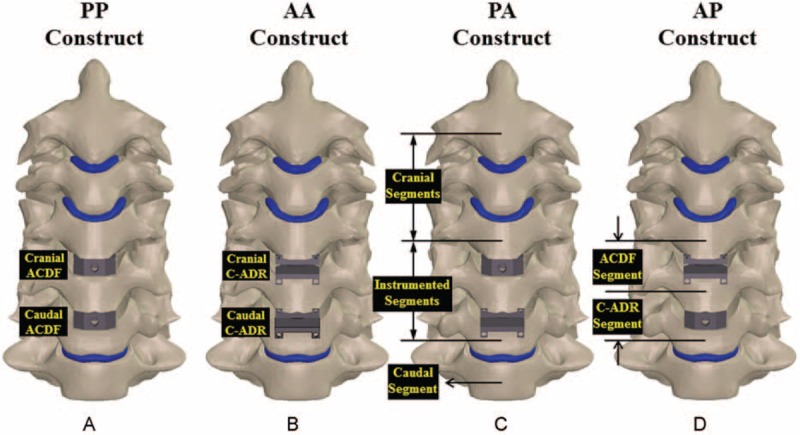
Four HS strategies are compared: (A) PP, (B) AA, (C) PA, and (D) AP constructs. All predicted results are normalized with the corresponding data of the intact construct.

The C4–C6 segments were modeled as moderate degeneration with the height reduced by 33%, the annulus area expanded by 40%, the nucleus modulus increased by 66%, and the facet gap decreased by 0.3 mm due to dehydration (Fig. 1).^19^ In terms of the 4 variations of hybrid strategies investigated in this study (Fig. 2), the C4–C6 segments were instrumented by peek cages and artificial discs for the PP and AA constructs, respectively. For the PA construct, the C4/C5 segment was fused by a peek cage and an artificial disc was instrumented into the C5/C6 segment. For the AP construct, the placement sites of artificial disc and peek cage were at the C4/C5 and C5/C6 segments, respectively. The peek cage and artificial disc were from the Cervios system (Synthes Inc, Paoli, PA) and Prestige LP Cervical Disc System (Medtronic Sofamor Danek Inc, Memphis, TN). The spikes of the cages were neglected for computational efficiency. A neurosurgeon monitored the placement of the artificial disc and peek cages. The terms “cranial” and “caudal” denoted the different cages and adjacent segments, respectively (Fig. 2C and D).

### Finite-Element Analyses

The bottom surface of the C7 vertebral body was fully constrained and the cervical column was flexed by the follower and concentrated loads (Fig. 1). The follower loads (73.6 N) were used to simulate the muscular contractions and stabilize the cervical column.^18^ The follower loads were activated by the tube–slider–cable mechanism that the slider could freely slide along the tube hole, while the springs were connected piece-wise by the sliders. The tubes were placed at the optimal sites posterior to the center of each vertebral body.^20^ The pulling load was exerted at the cable end tangential to the cable curve. The concentrated loads (1.0-Nm moment) were driven from head weight and muscular contractions and applied at the cervical top.^18^ Using the displacement-control method,^21^ the criterion for controlling the same motion of cervical constructs was adapted as a reasonable approach to evaluate the implant-induced effects on the adjacent segments and implants.

The interfaces of facet joints and artificial discs were modeled as the surface-to-surface contact elements that allowed separation and slippage, and excluded friction.^16,17^ The other interfaces between the implants and tissues were assumed to be bonded. Also, all implant materials were assumed to have linearly elastic, homogeneous, and isotropic properties throughout (Table 1). The calculated von Mises stresses of all implants were compared to the yielding strength of the corresponding material to validate the assumption of linear elasticity.

An automatic algorithm was used to generate the 10-node tetrahedral solid elements to mesh the cervical constructs. The mesh refinement was locally controlled at the high stress-concentrated sites and articulating surfaces. Using aspect ratio and the Jacobian check, the quality of all elements was monitored to avoid sharp discontinuities and unrealistically high stress concentrations. Mesh refinement was performed for modeling accuracy until excellent monotonic convergence behavior, with <5% difference in the total strain energy achieved. The nonlinear algorithm with large-deformation formula and direct-sparse solver was used by the software Simulation Ed. 2013 (SolidWorks Corporation, Concord, MA).

## VALIDATION OF THE FINITE-ELEMENT MODEL

Experimental and numerical comparisons were used to validate the simplifications and assumptions of the current model. Using the experimental and numerical data of the study by Kallemeyn et al,^17^ the assumed loads (1.0 Nm) were exerted onto the cervical top of the C2-C7 model to calculate the cervical range-of-motion (ROM), that is, total disc angle, of the current model. The calculated results were validated by the total disc angles for flexion, extension, bending, and rotation. For the facet forces, the current C3–C6 model was validated by extension data of the study of Jung et al.^18^ During validation, the initially chosen elastic moduli of the disc and some ligaments were slightly modified within the physiologic range to improve the agreement with the cadaveric results.

Five indices were chosen to evaluate the effects of the hybrid strategy on the adjacent tissues and implants. These were disc angle, disc stress, facet force, cage stress, and the stress and articulation of artificial disc. The von Mises stress was used as the index of the equivalent stress in this study. The disc and cage stresses were defined as the average value of the stresses within the overall disc and cage, respectively. The facet force was the sum of normal contact at the right and left facet joints. After flexion, the articulation of the artificial disc was defined as the relative slippage of the articulating surfaces.

Ethical approval: None. Because this study used the numerical method with finite-element analysis to evaluate the biomechanical differences between the hybrid instrumentation using fusion cage and artificial disc. But the patient consent was given for providing CT images.

## RESULTS

### Validation of the Finite-Element Model

**FIGURE 3:**
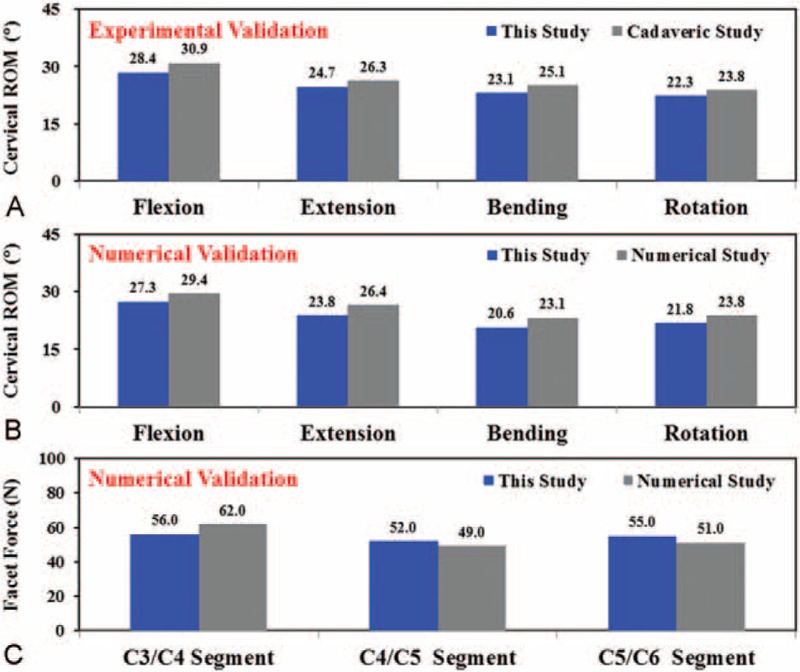
The predicted results of the current study are validated by cervical ROM and facet forces in the literature. A, Comparison with cadaveric results. B, C, Comparison with numerical results.

After slightly modifying the material properties of the discs and ligaments, a good agreement was achieved for the current and literature results (Fig. 3). Compared with the cadaveric study,^16^ the ROM error of this study was 8.8%, 6.5%, 8.7%, and 6.7% for flexion, extension, bending, and rotation, respectively (Fig. 3A). The predicted cervical ROMs for all levels fell within 1 standard deviation of the cited data.^16^ For the numerical validation,^16^ the cervical ROM error was 7.7%, 10.9%, 12.1%, and 9.2% for flexion, extension, bending, and rotation, respectively (Fig. 3B). The average error of the cervical motions was 7.7% and 9.9% for the experimental and numerical validation, respectively, which indicated good agreement and validated the current model. For the numerical validation,^16^ the error of the predicted facet forces was 10.7%, 5.8%, and 7.3% for C3/C4, C4/C5, and C5/C6 segments, respectively (Fig. 3C).

## TISSUE RESPONSES

**FIGURE 4:**
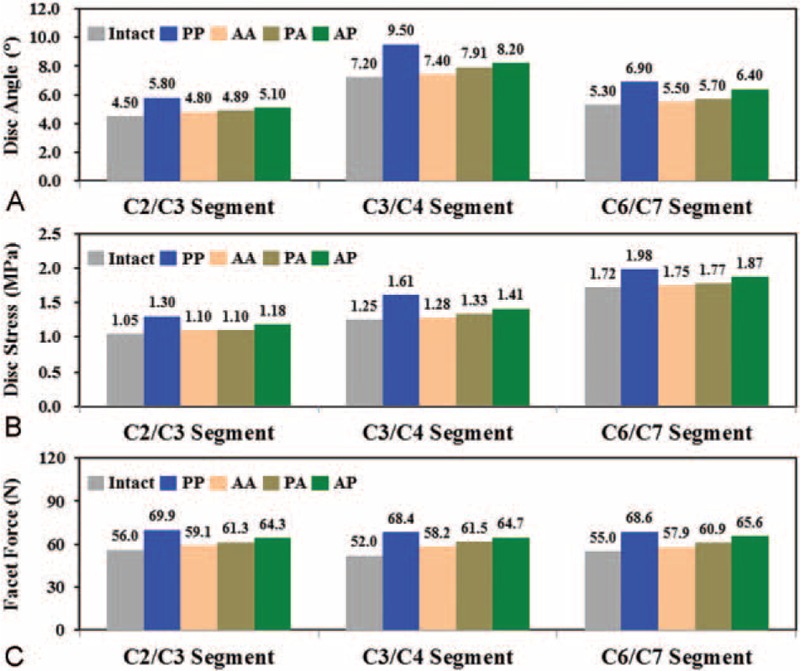
The predicted kinematic and mechanical results of the adjacent segments. A, Disc angles. B, Disc stresses. C, Facet forces.

**FIGURE 5:**
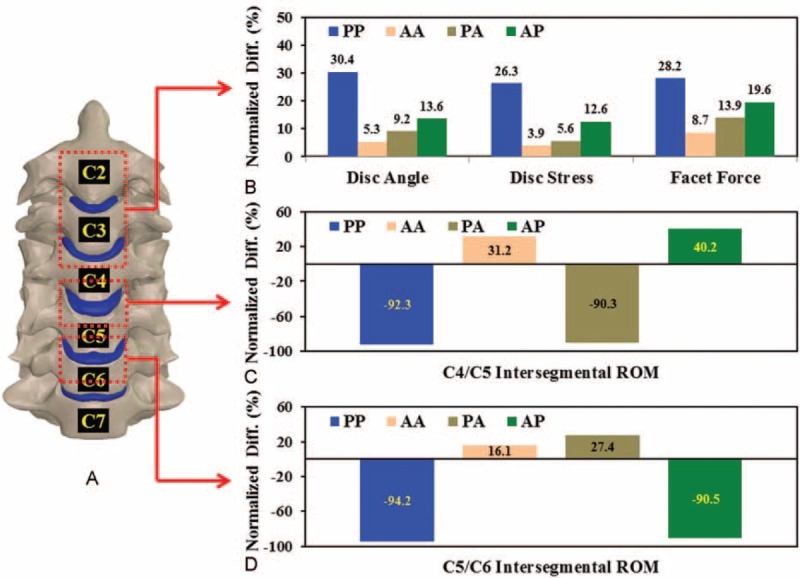
Comparison between normalized results of the cranial and instrumented segments. A, The averaged kinematic and mechanical results of the C2–C4 segments. B, C, The intersegmental ROMs of the C4/C5 and C5/C6 segments for the 4 constructs.

The effects of the different HS strategies were evaluated by the original and normalized results of the responses of adjacent tissue (Figs. 4 and 5). Normalization of the original data provided further insight into the differences between the instrumented and intact constructs. For the original data, the PP construct showed the highest limited mobility and concentrated load to the adjacent segments, followed by PA and AP. The AA had the least limited mobility and concentrated load (Fig. 4). At the C3/C4 segment, 2 stacked ACDFs increased disc angle by 31.9%, disc stress by 28.8%, and facet force by 31.5% compared with the intact construct. When 2 C-ADRs were stacked, the corresponding increases were only 2.8%, 2.4%, and 11.9%. Toward the C2/C3 and C6/C7 segments, the kinematic and mechanical compensation for the instrumentation also slightly decreased.

The kinematic and mechanical responses of the cranial and instrumented segments were normalized to the corresponding results of the intact construct (Fig. 5). For the 2 cranial segments, the normalized differences between the 4 strategies were consistent with the trends of the original data (Fig. 5A). The PP construct showed the most severe ASD progression, followed by the AP, PA, and AA. The increases in the normalized results of the PP construct were 5.7- (disc angle), 6.7- (disc stress), and 3.2-times (facet force) than those of the AA construct.

The instrumentation affected the intersegmental ROMs of the C4–C5 and C5/C6 segments (Fig. 5C and D). About 91% of the ROM of the fused segment was substantially reduced. Moreover, the C-ADR segment flexed much more (about 36%) than the intervertebral disc of the intact construct. At the C4/C5 segment, the C-ADR articulation increased the mobility of the AA and AP constructs by 31.2% and 40.2%, respectively. The C5/C6 fusion induced a 9.0% compensation for the increased articulation of the C4/C5 C-ADR (Fig. 5C). Similarly, the ACDF of the C4/C5 segment increased the C-ADR ROM of the C5/C6 segment by 11.3% for the PA construct (Fig. 5D).

### Implant Behaviors

**FIGURE 6:**
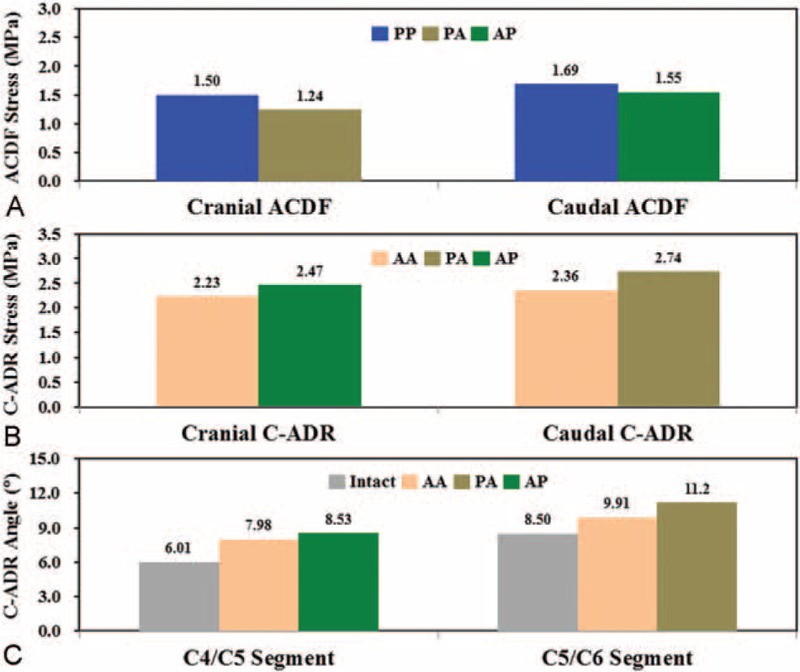
The stresses and angles of the peek cages and artificial discs. A, ACDF stresses. B, C-ADR stresses. C, C-ADR angles.

The mechanical and kinematic results of the ACDF and C-ADR implants are shown in Fig. 6. The rotary C-ADR consistently reduced the cranial ACDF stress by 17.3% for the PP versus PA construct and the caudal ACDF stress by 8.3% for the PP versus AP construct (Fig. 6A). However, the rigid ACDF made the cranial (for the AP construct) and caudal (for the PA construct) C-ADR stresses 10.8% and 16.1% higher than those of the AA construct (Fig. 6B). The artificial discs of the AA, PA, and AP strategies consistently showed concentrated articulation at the C-ADR interfaces compared with the intact disc (Fig. 6C). At the C4/C5 segment, the C-ADR angles of the AA and AP constructs increased by 32.8% and 41.9%, respectively. The C-ADR angles of the AA and PA constructs were 16.6% and 31.8% higher than the intact C5/C6 segment, respectively.

## DISCUSSION

Assuming the ACDF segment is a solid fusion, this study shows definite limitations in the intersegmental motion of the fused segment (Figs. 4 and 5C, and 5DFigures 4A, 5C and D). The loss in mobility of the fused segments increases the kinematic and mechanical demands of the adjacent discs and facets (Figs. 4 and 5BFigures 4B, C, and 5B). Such fusion-induced complication is well reported as the ASD problem by means of clinical, experimental, and numerical methods.^1–14^ However, even with C-ADR, all HS strategies increase the motion and load of the adjacent segments, especially for the PP construct (Fig. 5B). In general, the ACDF of the PA and AP constructs increase by an average of 10.0% the C-ADR ROM compared with the AA construct. This indicates that the C-ADR actually provides mobility-buffering ability to accommodate the compensated mobility of the ACDF segment (Fig. 5C and D).

The AA strategy shows minimal ASD, followed by PA and AP. The PP is the worst in inducing ASD (Fig. 5B). For the PP, PA, and AP strategies, the reported results are consistent with those of the study by Shin et al, which show that HS is better than 2-ACDF in terms of fewer postoperative neck pain, earlier C2–C7 ROM restoration, less adjacent ROM increase, and better neck disability index (NDI) improvement.^1^ This is one of the reasons for the higher mobility of 2 C-ADRs than 2 ACDFs. At the cranial segments, the normalized ROM between the PA and AP constructs are significantly different (Fig. 5B), indicating a significant effect of the placement strategies on adjacent segments.

The C5/C6 segment behaves as the most flexible segment. In a review paper, Lee et al reported that the hybrid use of ACDF might lead to heterotopic ossification due to increased loads onto the C-ADR.^2,3,9^ The current study predicts consistent results that both the PA and AP strategies increase C-ADR stresses (about 13.4%) and angles (about 10.0%) (Fig. 6B and C). In contrast, the C-ADR articulation can provide about 30.8% higher ROM than the corresponding disc of the intact construct (Fig. 6C). In this study, the follower loads and cranial moment make the cervical column flex 4.5°, 7.2°, 6.0°, 8.5°, and 5.3° from the cranial to the caudal segments. The placement of the ACDF at highly flexible segments leads to higher ASD compensation from the C5/C6 to the adjacent segments. This accounts for the worse ASD progression of the AP compared with the PA constructs (Figs. 4 and 5). This provides surgical information that the physiologic motion of the C5/C6 segment should be preserved by using the C-ADR rather than the ACDF.

As with any numerical model that attempts to simulate cervical complexity, there are some limitations and underlying assumptions inherent in this study. Limited by the data source, this study does not include degenerative changes due to vertebral osteoporosis, facet osteoarthritis, endplate sclerosis, and annular tears. Their effects on the predicted results are not investigated independently and systematically. This study also shows the minimal ASD progression by the AA strategy than the others. Regarding complication, however, a remarkably lower rate of dysphagia is documented after a single-level C-ADR compared with a multilevel C-ADR at 2 years of follow-up.^2^ Barbagallo et al also report that the radiologic outcome of heterotopic ossification may be present for the C-ADR group up to 8.3% after 67.2-month follow-up.^3^ Last, surgical factors such as C-ADR placement, interfacial micromotion, and heterotopic ossification are not considered in the simulation.

## CONCLUSION

**FIGURE 7:**
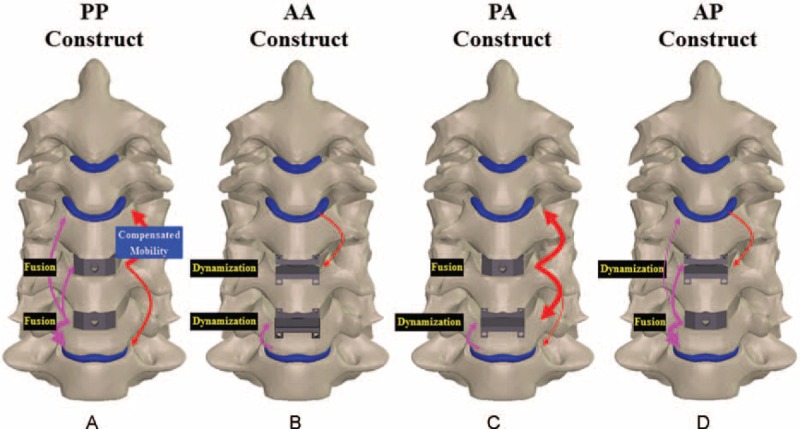
Mobility-buffering mechanisms to schematically illustrate the compensated mobility of ACDFs and C-ADRs of the 4 constructs. The (A) PP, (B) AA, (C) PA, and (D) AP constructs.

The ACDF definitely increases the kinematic and mechanical demands of the adjacent segments, whereas the C-ADR articulation serves as a mobility buffer of the ACDF segment to depress ASD progression (Fig. 7). Consequently, the AA and PA can preserve the mobility of the most flexible C5/C6 segment. Thus, they are recommended as the HS strategies of the two-level instrumentation. If surgical cost is a major concern, the PA is a tradeoff strategy to preserve mobility and reduce cost. The AP can be used in situations where cranial segments are initially degenerated but not treated in the current stage. However, the AP strategy still induces less ASD problem than the PP treatment.
